# Comparative study of conventional urothelial carcinoma, squamous differentiation carcinoma and pure squamous carcinoma in patients with invasive bladder tumors

**Published:** 2014-06-25

**Authors:** G Gluck, M Hortopan, D Stănculeanu, M Chiriță, R Stoica, I Sinescu

**Affiliations:** * Department of Surgical Urology, Dialysis and Renal Transplantation, "Fundeni" Clinical Institute, Bucharest; ** Department of Pathology, "Fundeni" Clinical Institute, Bucharest; *** Department of Medical Oncology, "Alexandru Trestioreanu" Oncologic Institute, Bucharest

**Keywords:** urothelial, squamous, squamous differentiation

## Abstract

Abstract

Purpose: Treatment results evaluation (radical cystectomy and adjuvant chemo/radiotherapy) in patients with urothelial carcinoma, squamous differentiation carcinoma and pure squamous bladder carcinoma.

Material and methods: The study included 361 patients with invasive bladder carcinoma treated between 1990-2013. Histology showed 296 cases of urothelial carcinoma (82% - group A), 52 cases of urothelial divergent differentiation (squamous and urothelial carcinoma 14.4% - group B) and 13 cases of squamous cell carcinoma (3.6% - group C). All patients benefited from radical cystectomy. Adjuvant chemotherapy was undergone in 68 patients.

Results: Group A - urothelial carcinoma - had a 44% rate of patients alive with a mean survival period of 73 months. About 56% of the patients died, the mean survival period being 4 years.

Group B – urothelial carcinoma with squamous differentiation – had a mean survival period of 36 months (between 1-156 months). 17 patients (33%) are alive at 50 months postoperatively.

Group C – squamous carcinoma – had a mean survival period of 9.4 months.

Discussions: Locally advanced disease was diagnosed in 50% of the patients in group A, while in group B the rate was 84.6% and 70% in group C, respectively.

Conclusions: Squamous pattern detected in the histopathological specimen represents a negative prognostic factor. It seems that the squamous component influences the outcome of the disease due to its biological characteristics in the evolution of squamous carcinoma, with advanced local stage disease at diagnosis – late onset of symptoms and lack of response to adjuvant treatment.

Abbreviations: SCC = squamous cell carcinoma; MSK = Memorial Sloan Ketering

## Introduction

Bladder cancer can be classified as conventional urothelial carcinoma, divergent differentiation urothelial carcinoma and non-urothelial carcinoma. Statistically, 90-95% of all bladder cancers are urothelial carcinomas, while the other 5-10% encompass epithelial and/or mezenchymal tumors. The association between urothelial and other histological features (squamous cell carcinoma, adenocarcinoma, micropapillary carcinoma [**30**], neuroendocrine carcinoma) define divergent differentiation [**1,29**]. Assessment in such cases is made by reporting the percentage of the associated pattern – focal (<10%), intermediate (<50%) or extensive (>50%). Squamous cell carcinoma is a relatively rare disease, responsible for 1.4 – 4.5% of all newly diagnosed bladder cancers in USA and Western Europe, mostly in the 7th decade of life. Male female ratio found in literature is 1.25:1/1.8:1. By definition, squamous cell bladder carcinoma does not include urothelial features. The main histological characteristic is the presence of keratin forming cells that have a pearly aspect and intercellular bridges (Qihui). It is well known that each histological variant has a different biology, subsequently possessing an unique capacity of recurrence, metastasization and treatment response (P Black Urologic Oncology 2009). Therefore, these features must guide, influence and change the standard treatment protocol.

## Materials and methods 

The study included 361 patients with invasive bladder carcinoma treated between 1990 and 2013. Histology showed 296 cases of urothelial carcinoma, 52 cases of urothelial divergent differentiation (squamous and urothelial carcinoma) and 13 cases of squamous cell carcinoma (**Table 1**).

**Table 1:**
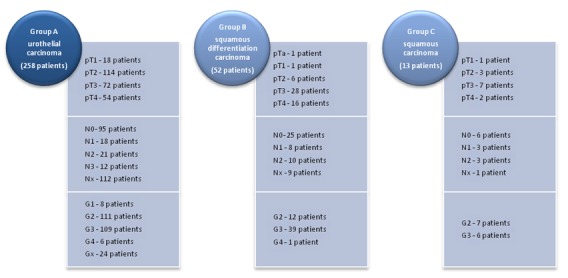
Stratification of the 361 patients with invasive bladder tumors according the pathological findings

In group A, 39 patients were lost to follow up. The mean age in group A was 61 years, the group including 18 females and 240 males. The TNM staging reported 18 cases of pT1 disease, 114 cases of pT2, 72 cases of pT3 and 54 cases of pT4; 95 patients had N0, 18 patients N1, 21 patients N2 and 12 patients N3, with no lymphadenectomy undergone in 112 patients. Metastases were present at the moment of diagnosis in 8 patients. Tumor grading showed 8 cases of G1, 111 cases of G2, 109 cases of G3, 6 cases of G4 and 24 cases were tumor grading that could not be assessed.

Group B comprising of 44 males and 8 females, had a mean age of 61.7 years (between 44-78 years). Tumor staging included 1 case of pTa, 1 case of pT1 6 cases of pT2, 28 patients of pT3 and 16 patients of pT4; 18 cases of positive lymph nodes (N1-8 cases, N2- 10 cases), 25 cases of negative lymph nodes and 9 cases without lymph node dissection. Tumor grading included 12 cases of G2, 39 cases of G3 and 1 case of G4. Liver metastasis was found in only one patient.

Squamous cell carcinoma was detected in 13 male patients - group C. The mean age was 59 years, the group including 1 case of pT1, 3 cases of pT2, 7 cases of pT3, 2 cases of pT4, 6 cases of N0, 3 cases of N1, 3 cases of N2 and 1 case of Nx. No metastases were found at the moment of diagnosis. Tumor grading was G2 in 7 cases and G3 in 6 cases.

## Results

 In the urothelial carcinoma group, 44% of the patients are alive with a mean survival period of 73 months. The rest (56%) died with a mean survival period being 4 years. 

 Group B – urothelial carcinoma with squamous differentiation – had a mean survival period of 36 months (between 1-156 months). 17 patients (33%) are alive at 50 months postoperatively. 

 Group C – squamous carcinoma – had a mean survival period of 9.4 months. From the total of 13 patients, 2 were lost to follow up, 2 are alive at 7 and 48 months, respectively (mean survival period – 27.5 months). 9 patients died at an interval which varied between 1-24 months postoperatively (mean survival period – 9.4 months). Cause of death was identified in 8 patients: local recurrence in patients with R2 disease (4 cases), liver metastasis (2 cases) and sepsis after ureterosigmoidostomy (2 cases). Almost 70% of the patients were diagnosed with locally advanced disease. 

 Adjuvant treatment 

 The study group was composed of 68 patients with invasive bladder cancer (51 patients – urothelial carcinoma, 12 patients - urothelial with squamous differentiation carcinoma, 5 patients – squamous carcinoma) treated between 1990-2010. Adjuvant treatment was given accordingly to the histopathologic exam (chemo +/- radiotherapy). 

 Urothelial carcinoma was diagnosed in 50 males and 1 female, mean age of the group being 59 years. Staging reported 2 cases of pT1, 16 cases of pT2, 21 cases of pT3, 12 cases of pT4, 18 cases of N0, 10 cases of N1, 10 cases of N2, 2 cases of N3 and 11 cases of Nx. Tumor grading revealed 2 cases of G2 and 11 cases of G3. 

 12 patients with divergent differentiation (urothelial and squamous carcinoma) received the indication for adjuvant treatment accordingly to the TNM-G staging: pT1 – 1 case, pT2 – 2 cases, pT3 – 4 cases, pT4 – 5 cases, N0 – 2 cases, N1 – 2 cases, N2 – 5 cases, N3 – 1 case, Nx – 2 cases, G2 – 2 cases, G3 – 11 cases. 

 The squamous carcinoma group included 5 male patients with a mean age of 56 years. TNM-G staging was as it follows: pT1 – 1 case. pT2 – 1 case, pT3 – 2 cases, pT4 – 1 case, N0 – 1 case, N1 – 2 cases, N2 – 1 case, Nx – 1 case, G2 – 3 cases, G3 – 2 cases. 

 The results showed a mean survival period in the urothelial group of 37 months. 8 patients are alive with a mean survival period of 88 months (16-216 months). 41 patients died at 2 – 156 months after diagnosis (5 non-oncologic deaths), with a median survival period of 27 months. Disease related death was reported in 29 patients: by local recurrence [**16**] or distant metastasis [**13**]. Cause of death could not be identified in 7 cases. 2 patients were lost to follow up. 

 In the mixed urothelial carcinoma group the mean survival was 33 months. From the total number of 12 patients, 3 are alive with a mean survival period of 71 months, 8 patients died (mean survival period – 18 months) and one was lost to follow up. 

 In the squamous carcinoma group, the mean survival period was 17.2 months.

## Discussions

Squamous cell bladder carcinoma is a rare entity, accounting for less than 3.6% [18,23], of all bladder cancers newly diagnosed. The most frequently encountered risk factors are black race [**22**], bladder diverticuli [**20**], chronic urinary infections [**2**], indwelling catheters (Stonchill), history of cyclophosphamide usage [**28**], intravesical BCG instillations [**14**], male gender (Lynch 1995), smoking [**2**], bladder stones and foreign bodies [**25**]. Human papilloma virus does not induce squamous bladder cancer. 

 Taking into consideration the etiology, squamous bladder carcinoma is divided into bilharzial type, commonly found in North Africa and nonbilharzial type, caused by chronic urinary infections, bladder stones, etc. [**24**], 

 Messing reports in a study on a cohort of 1422 patients with nonbilharzial SCC diagnosed between 1988-2003 from SEER database, a mortality rate of 11% and 72%, respectively at 2 years for stage I and IV disease. After adjustment for age, gender, race and initial treatment, squamous cell carcinoma was associated with poor outcome when compared to urothelial cancer in patients that did not benefit from radical cystectomy in stage I and II and also for all patients (with or without cystectomy) in stage III and IV disease. 

 Comparative studies with urothelial carcinoma are causing controversy [**1,8,11,19,21**],. Craig analyzed 67 patients with nonurothelial carcinomas who represent 7% from a cohort of patients with invasive bladder cancer treated by radical cystectomy, showing that the 5-year survival rate for SCC was 55% vs 60% for transitional cell carcinoma. 

 Another recent study from MSK (Behfar 2012) which included 2031 patients with radical cystectomy, reported 78 cases of SCC and 67 cases of squamous differentiation. No major differences regarding survival are seen between the two groups. 

 Radiotherapy alone offers a 5-year survival rate of 12-31% [**3,4,10,12**]. Rundle [**23**], shows that the 1-year survival rate is 23.8%, while the 5-year survival rate is 1.9% in a study undergone between 1964 and 1978 on a cohort of 114 patients with SCC treated by radiotherapy. 

 Some studies argue in favor of neoadjuvant radiotherapy, but without any controlled studies [**5,6**]. 

 In 1989, Swanson performed neoadjuvant radiotherapy (50Gy) in 25 patients who benefited from radical cystectomy (1951-1985). The results showed 25% [**10**] of patients with partial tumor regression and 24% of patients with total tumor regression - T0. No pelvic lymphadenectomy was performed in 20 patients. The 5-year survival rate was 50%, with 43% for T3 and T4 stages. 

 In 2007, Kassouf [**17**], published a study consisting of 27 cases of SCC (1988-2003) with a 2 year survival rate of 47.6% and a recurrence free survival rate of 32.8%. 8 patients received neodjuvant chemo +/- radiotherapy. Due to disease progression, 5 patients did not fit for radical cystectomy. The other 3 patients showed disease regression in 2 cases after neodjuvant treatment and did not develop recurrences. 10 patients with radical cystectomy developed recurrences at a mean period of 5.1 months, 7 patients dying due to local recurrence (3 cases), distant metastasis (1 case) or both (3 cases). Median survival period, disease specific survival period and recurrence free survival period was estimated on Kaplan-Meier to be of 12.4 months, 12.4 months and 8.3 months respectively. 

 Squamous cell bladder carcinoma seems to have no response at chemotherapy [**24,27**]. Despite this fact, Glasky [**7**], published a study with complete response at administration of isophosphamide, paclitaxel and cisplatin in 2 out of 8 patients. 

 Data from literature reveals a low incidence of metastatic disease in SCC – 8% [**26**]. Jones confirms such facts in a study on 51 patients with SCC showing that death event occurred by failure of local disease control rather than by distant metastasis. 

 In contrast to that, urothelial carcinoma tends to have local recurrences in 50% of the cases, while 50% associate distant metastasis with local recurrence [**15**]. 

Therefore, the need for a better pelvic control with negative surgical margins in the cystectomy specimen arises [**26**]. 

In our study, the response to adjuvant treatment must be correlated with the locally advanced disease at diagnosis (65% in urothelial carcinoma), tumor grading (78% - G3 and G4) and R2 disease (7 patients). 

 The squamous differentiation group recorded 75% of patients [**9**] with locally advanced disease, 91% having G3, while the SCC group recorded 60% of patients with locally advanced disease. 

 The most aggressive tumors tend to be the SCC. Better results regarding survival are observed in patients who receive adjuvant treatment compared to cystectomy alone (17.2 months vs 6 months). 

## Conclusions

Standard treatment for nonbilharzian SCC is radical cystectomy, offering local control for the disease. A feature of SCC seems to be advanced local disease – 70% of patients. Therefore, death occurs more frequently by local recurrence rather than by distant metastasis. Neoadjuvant radiotherapy can be taken into consideration, as SCC is radiosensitive. Though data suggest that chemotherapy has no indication in SCC, in our study higher survival periods were achieved in the chemotherapy group. 

 Concerning the aggressiveness of these pathologies we can conclude that squamous carcinoma is the most aggressive, followed by squamous differentiation carcinoma with a median survival period of 33 months (lower than 37 months) and urothelial carcinoma – 37 months median survival (less than 4 years). 
